# Evaluation of the Antiviral Potential of Modified Heterocyclic Base and 5’-Norcarbocyclic Nucleoside Analogs Against SARS-CoV-2

**DOI:** 10.32607/actanaturae.11479

**Published:** 2021

**Authors:** E. S. Matyugina, M. S. Novikov, L. I. Kozlovskaya, V. P. Volok, E. Y. Shustova, A. A. Ishmukhametov, S. N. Kochetkov, A. L. Khandazhinskaya

**Affiliations:** Engelhardt Institute of Molecular Biology, Moscow, 119991 Russia; Volgograd State Medical University, Volgograd, 400131 Russia; FSBSI “Chumakov Federal Scientific Center for Research and Development of Immune and Biological Products of the Russian Academy of Sciences”, Moscow, 108819 Russia; Sechenov Moscow State Medical University, Moscow, 119991 Russia

**Keywords:** SARS-CoV-2, antiviral drugs, nucleosides, nucleoside analogs

## Abstract

The pandemic caused by the novel betacoronavirus SARS-CoV-2 has already claimed more than 3.5 million lives. Despite the development and use of anti-COVID-19 vaccines, the disease remains a major public health challenge throughout the world. Large-scale screening of the drugs already approved for the treatment of other viral, bacterial, and parasitic infections, as well as autoimmune, oncological, and other diseases is currently underway as part of their repurposing for development of effective therapeutic agents against SARS-CoV-2. In this work, we present the results of a phenotypic screening of libraries of modified heterocyclic bases and 5’-norcarbocyclic nucleoside analogs previously synthesized by us. We identified two leading compounds with apparent potential to inhibit SARS-CoV-2 replication and EC_50_ values in a range of 20–70 μM. The structures of these compounds can be further optimized to develop an antiviral drug.

## INTRODUCTION

Coronaviridae is a viral family that comprises two subfamilies: Orthocoronavirinae and Letovirinae. The Orthocoronavirinae subfamily includes dangerous human pathogens. Human coronaviruses (HCoVs) HCoV-OC43 (OC43) and HCoV-229E (229E) were first identified in the 1960s [1]. Later, other human coronaviruses were discovered: HCoV-NL63 (NL63) in 2004, and HCoV-HKU1 (HKU1) in 2005 [1]. These four viruses usually cause acute diseases of the upper and (less often) lower respiratory tract, but a severe coronavirus infection is diagnosed rarely and is usually considered to be due to a concomitant pathology and/or immunological aging. Two more pathogenic human coronaviruses, SARS-CoV (2003) and MERS-CoV (2012), cause atypical symptoms of the upper and lower respiratory tract, which are especially severe in people over 65 years of age and patients with comorbidities [1].

SARS-CoV-2, which was identified in Wuhan city (China) in December 2019, is seventh in the group of human coronaviruses. The infection caused by this virus, which is called COVID-19, spread rapidly around the world. The World Health Organization announced the COVID-19 pandemic on March 11, 2020 [2]. The combined efforts of researchers around the world, which made it possible to quickly identify the etiological agent and obtain information on the virus structure and life cycle, as well as to develop agents for the treatment of the atypical pneumonia caused by SARS-CoV, led to the emergence of vaccines, some of which have successfully passed preclinical and clinical trials and are now used for mass vaccination [2].

Despite that, a COVID-19 diagnosis had been confirmed in more than 274 million patients in the world by October 27, 2021; of these, 4.96 million people had died [2]. To date, there are no generally accepted effective strategies to treat COVID-19. For this reason, the creation of specific drugs against this disease remains topical. Therapeutic agents based on antibodies, inhibitors of viral enzymes (RNA-dependent RNA polymerase, proteases, etc.), inhibitors of viral entry into the cell, etc. are currently being developed. Intensive research of drugs for treating other viral (influenza, HIV infection, hepatitis C, Ebola, etc.), bacterial, and parasitic infections, as well as autoimmune, oncological and other diseases, is currently underway as part of repurposing of approved drugs [3].

We screened the library of heterocyclic bases and nucleoside analogs with antiviral [4, 5, 6, 7, 8], antibacterial [7, 9, 10, 11], antiparasitic [12, 13], and antitumor [14, 15] activities.

## EXPERIMENTAL

Stock 5 μM solutions of test compounds in 100% dimethyl sulfoxide (DMSO) were prepared. The SARS-CoV-2 virus strain PIK35 (GISAID ID EPI_ISL_428851) [16] was used to assess the antiviral activity of the compounds. The virus was passaged five times in Vero cells and stored as an infected cell suspension at –70°C. African green monkey kidney Vero cells were received from Biologicals (WHO, Switzerland; RCB 10-87). The cells were maintained in a DMEM medium (Chumakov Federal Scientific Center for Research and Development of Immune-and-Biological Products of the Russian Academy of Sciences, Russia) with 5% fetal bovine serum (Gibco, USA), 0.1 mg/ml streptomycin, and 100 U/ml penicillin (PanEco, Russia).

The phenotypic screening method [16] was used. Eight two-fold dilutions of compound stock solutions in a DMEM medium were prepared. The compound dilutions were then mixed with equal volumes of the viral suspension containing 50–200 TCID50 per well and incubated at 37°C for 1 h. The virus–compound mixture was added to confluent Vero cell monolayers in duplicates. After 5-day incubation at 37°C, the cytopathic effect (CPE) was assessed using a microscope. EC_50_ values were calculated using the Karber method as previously described [16]. The experiment was repeated at least two times for each compound. N(4)-hydroxycytidine (NHC) and DMSO were used as a positive and negative control, respectively.

## RESULTS AND DISCUSSION

**Fig. 1:**
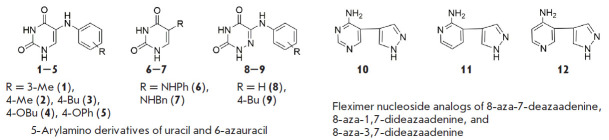
Heterocyclic base analogs

**Fig. 2:**
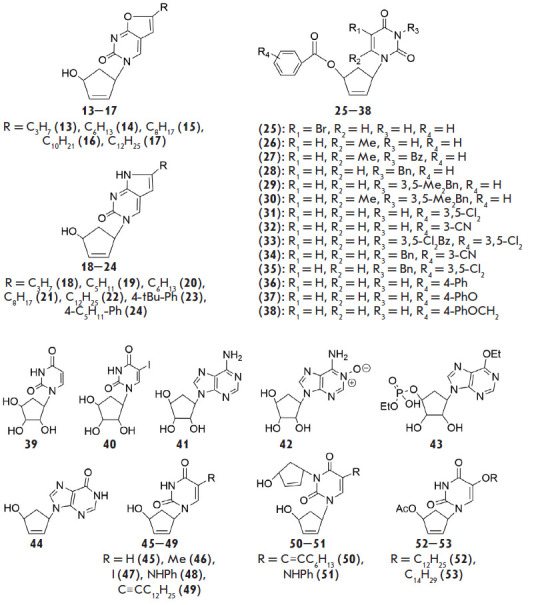
5’-Norcarbocyclic nucleoside analogs

The library of heterocyclic base (Fig. 1) and nucleoside (Fig. 2) analogs, previously synthesized by us, was screened phenotypically for activity against SARS-CoV-2. The first group of heterocyclic base analogs is 5-arylamino derivatives of uracil and 6-azauracil (Fig. 1), which were shown to act as non-nucleoside inhibitors of HIV and inhibitors of Mycobacterium tuberculosis growth [7, 11]. The second group includes new fleximer analogs of aza/deazapurine bases (Fig. 1) [17]. Aza/deazapurines, as well as the appropriate nucleosides, are known to exhibit a wide range of antiparasitic, antitumor, and antiviral properties [18]. At the same time, fleximer bases happen to exhibit high structural mobility (flexibility), which is due to a splitting of the purine ring into separate heterocyclic fragments. Free rotation around the C–C bond allows these compounds to better accommodate to the spatial structure of the target enzyme active site, which in some cases enables them to bypass point mutations in the enzyme, thus providing a mechanism that helps to avoid drug resistance [17].

Another group of compounds includes 5’-norcarbocyclic analogs of purine and pyrimidine nucleosides (Fig. 2). The structural feature of these analogs is that they lack the 5’-methylene group. Because of that lack, these compounds cannot be converted by cellular enzymes to phosphorylated derivatives and, thus, cannot exhibit biological activity in reactions typical of conventional modified nucleosides. However, representatives of this class of compounds can act as HIV non-nucleoside reverse transcriptase inhibitors (NNRTIs) and also exhibit antibacterial and antitumor activities [4, 5, 6, 7, 9, 10, 11, 15].

The antiviral activity was recognized through the ability of the test compounds to inhibit Vero cell death induced by infection with the SARS-CoV-2 strain PIK35. A well-known inhibitor of SARS-CoV-2 replication, N(4)-hydroxycytidine (NHC), whose activity in this series of experiments was consistent with the previously obtained data, was used as a positive control [16]. A total of 53 compounds were tested, most of which showed no activity at concentrations < 100 μM. Only two compounds, namely with EC50 values of 53 and 21 μM, respectively (Table). They also exhibited a strong cytotoxic effect, which is consistent with the previously obtained data [14].

**Table 1:** Antiviral activity and cytotoxicity constants of active compounds

Compound	EC_50_, μM (M ± SEM)	CC_50_, μM (M ± SEM)	Selectivity index (SI)
23	53 ± 18	75 ± 25	1.42
24	21 ± 6	53 ± 18	2.52
NHC	5.3 ± 0.9	> 100	> 19

## CONCLUSION

In this work, we performed phenotypic screening and identified two nucleoside analogs that can inhibit SARS-CoV-2 replication in vitro: 5’-norcarbocyclic derivatives of bicyclic furano[2,3-d]pyrimidines **23 **and **24**. The structures of these compounds can be further optimized to develop an antiviral drug.

## Abbreviations

EC50: 50% effective concentration, i.e. the compound concentration that inhibits viral replication by 50%
NHC: N(4)-hydroxycytidine
SARS-CoV-2: Severe Acute Respiratory Syndrome Coronavirus 2
HIV: Human Immunodeficiency Virus
DMSO: dimethyl sulfoxide
COVID-19: coronavirus disease 2019
TCID50: 50% tissue culture infective dose, i.e. the viral dose that causes cytopathic effects in 50% of tissue culture cells.
